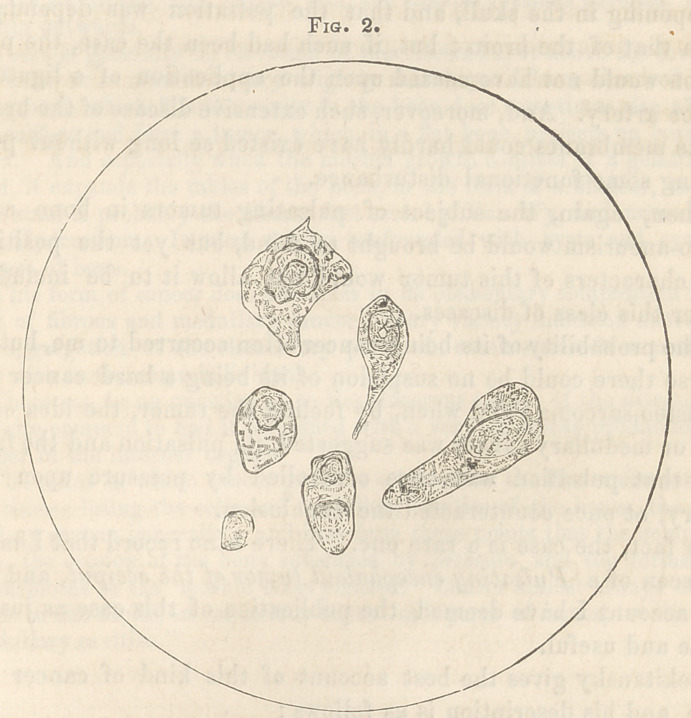# Case of Pulsating Tumor of the Occiput

**Published:** 1854-02

**Authors:** Jno. Neill

**Affiliations:** Surgeon to the Pennsylvania Hospital


### MEDICAL EXAMINER.

NEW SERIES.—NO. CX.—FEBRUARY, 1854.

### ORIGINAL COMMUNICATIONS.

*Case of Pulsating Tumor of the Occiput.* By JNO. NEILL, M. D., Surgeon to the Pennsylvania Hospital.

From the Notes of Dr. JAS. E. RHOADS, House Surgeon.

David Patterson, aged 70, was admitted into the Pennsylvania Hospital, April 28, 1853. He stated that for many years he had a small hard tumor upon the right side of the back part of the head, which never pulsated or gave him any pain till about five months previous to his admission, when accidentally pressing the tumor against the pillow whilst lying in bed he heard something crack in it, and that it had constantly enlarged since this occurrence.

At the time of his admission there existed a large, regularly rounded tumor upon the right posterior part of the head, commencing about three quarters of an inch behind the right ear, and extending to the left of the median line posteriorly. It reached also from the margin of the hairy scalp nearly to the top of the head. It was eight inches from side to side in either direction over the most prominent part, and sixteen inches in circumference around the base.

The accompanying wood-cut is taken from a very striking likeness of the patient taken by my friend Dr. F. W. Lewis.

The skin over the tumor was stretched and reddened, but not hot nor tender, and could be moved freely upon the parts beneath. There was no pain or uneasiness in the tumor, except a sense of tension.

It had a *pulsation* distinctly perceptible both to the eye and touch, accompanied by a marked *aneurismal bruit.* The pulsation was not a simple rising and falling of the tumor, but an expansion in all directions.

The right occipital artery could be felt beating strongly and with a distinct thrill. Pressure upon it sensibly diminished the pulsation of the tumor, and pressure upon both occipitals almost entirely destroyed pulsation.

There was no swelling of the glands in the vicinity, and no other tumor about the body. The pulse was regular—the radials were not ossified—and the sounds of the heart were natural. By the 30th, the tumor had rapidly increased in size, the skin over it became reddened and tense, and threatened soon to give way, and it was decided to tie both occipitals. Each vessel was secured on the cardiac side of the origin of the princeps cervicalis. After the operation no pulsation could be perceived, nor could the bruit be heard. The tumor became somewhat smaller and much less tense. Its color also was much less deep.

In the evening, however, the patient had some fever, and the pulsation returned strongly. On the following day, May 1st, the pulsation was nearly as strong as ever, but the bruit was scarcely audible; the tumor was hot and the skin over it red. Cold was applied by lint dipped in ice water.

*May* 3. The tumor was smaller, the pulsation decidedly less —no bruit, the skin less red—no fever. The wound looked well and had partially healed. The cold was continued and compression maintained by means of a bandage.

*May* 5. Pulsation was still distinct—the bruit just audible. A small abscess had formed in the left wound beneath the skin, which had united. The evacuation of the pus was followed by a chill and subsequent fever.

*May* 7. Erysipelatous inflammation attacked the tumor and spread over the whole scalp. The inflammation gradually extended over the face and a portion of the neck, and was attended with great swelling and severe general prostration.

Upon the 14th, the right ligature came away.

*May* 16. The erysipelas had disappeared, leaving the integuments of the tumor œdematous and much reddened. The pulsation remained about the same, but still somewhat less than before the operation. The tumor was covered with collodion daily, with reference to its contracting effect and the support it would afford to the skin.

*May* 21. The remaining ligature came away. There is little or no change in the size of the tumor or its pulsation. The patient's general health is as good as before the operation.

The collodion was constantly applied, and a roller so placed around the base of the tumor as to constrict it and press upon the small vessels supplying it. Small branches of the temporal arteries could be felt entering the tumor, and the posterior auriculars were enlarged. Pressure upon the temporals had no appreciable effect upon the pulsation.

He remained in the house until July 17, when he applied for his discharge, thinking himself sufficiently relieved to attend to some little business. When he left the Hospital the tumor was about the same size as on his admission, but the pulsation and bruit were much less. There was no pain or tension in it, and it showed no disposition to extend itself or to ulcerate. The skin over it was loose and could readily be moved upon the parts beneath.

In September he died at the Alms-house, and after the postmortem had been made I had an opportunity of examining a section of the head containing the tumor; it had encroached upon the cavity of the cranium, through an opening with rough and jagged edges, of about three inches in diameter.

The dura mater was pushed into the cranium and was closely connected by its external surface with the tumor. The internal surface of the dura mater seemed perfectly healthy.

Upon cutting into the tumor it presented the appearance of encephaloid cancer. The larger part of the section was of that white kind which so much resembles medullary matter. and the remainder had a pinkish grey tint, indicative of greater vascularity. The interior of the tumor was intersected with numerous dense bands, and in the intervals were several small cysts containing fluid.

About one inch and a half from the tumor there had been

Fig. 1.

disease and absorption of a portion of the parietal bone. The opening in the bone was one inch in diameter, and seemed to be so regularly circular on one side that it appeared to have been made with a trephine. The pericranium and the dura mater did not seem to be diseased, but between the two there was a reddish material, so soft that it was almost semifluid.

The microscopic examination of these products shows their true nature.

When the material of the tumor was first examined, fig. 1, (see preceding page,) the cancer cell was much obscured by oil globules, which were so numerous as to suggest the idea that the growth was undergoing fatty degeneration. The cells were pale, large, irregular in form, and frequently folded and wrinkled.

Fig. 2.

The nucleus was very large. Fig. 2 represents a field exhibiting the cells from the eroded spot in the cranium near the tumor, and very distinctly reveals the cancerous nature of the disease.

*Remarks.* The reader will probably be surprised at the manner in which the foregoing case terminated. There was certainly a great want of correspondence in the physical characters of the disease, and those revealed by the post-mortem examination.

Here was a pulsating tumor, with perfect aneurismal pulsation and bruit; pressure on the occipitals interrupted the pulsation, and the ligature subsequently destroyed the pulsation and bruit completely. The impression that it was an aneurism was irresistible, and I thought that it was a diffused aneurism. Subsequently, however, to the operation the pulsation returned, and doubts began to arise as to its aneurismal nature, still there was no reasonable grounds for such suspicions. Under such circumstances the attention of any one would naturally be directed to the possibility of its being a disease of the brain or dura-mater, which had worn an opening in the skull, and that the pulsation was dependant upon that of the brain; but, if such had been the case, the pulsation would not have ceased upon the application of a ligature to the artery. And, moreover, such extensive disease of the brain or its membranes could hardly have existed so long without producing some functional disturbance.

Then, again, the subject of pulsating tumors in bone and osteo-aneurism would be brought to mind, but yet the position and characters of this tumor would not allow it to be included under this class of diseases.

The probability of its being cancer often occurred to me, but of course there could be no suspicion of its being a hard cancer or an osteo-sarcoma; and when, by feeling the tumor, the idea of a soft or medullary cancer was suggested, its pulsation and the fact that that pulsation was once controlled by pressure upon an artery, at once counteracted the conclusion.

In fact, the case is a rare one. There is no record that I have yet seen of a *Pulsating encephaloid tumor of the occiput*, and on this account I have deemed the publication of this case as justifiable and useful.

Rokitansky gives the best account of this kind of cancer in bone, and his description is as follows:

"There is a peculiar form of cancer, which Otto describes as a gnawing or erosion of bone. Lobstein speaks of it under the title of Osteolyosis; but he includes amongst his cases some which were examples of cystoid disease and cystosarcoma, and perhaps also of areolar cancer. On the broad bones of the skull, or on the ossa innominata, spots are noticed in which a foreign substance occupies the place of the natural bone. Besides other peculiarities, this substance presents very various degrees of consistence, sometimes being lardaceo-cartilaginous, and white or whitish-red; sometimes a fleshy-fibred, red substance; sometimes a gelatinous, an albumino-serous, or a fatty and serous fluid of a yellowish or greyish color, or altogether colorless. It commences in the diplœ, which it soon eats away, forming a cavity which, in the bones which have been mentioned, is at first inclosed within their compact tables. This covering disappears at several points, and leaves a smooth round, an oval, or an irregular sinuous opening, or a gap, which is covered on both sides by periosteum. The morbid growth then interweaves itself with this membrane, especially with the dura mater when the skull is affected; and not unfrequently advances in it beyond the margins of the opening in the bone. The diplœ is usually eroded to a greater extent than the compact walls of the bone, and hence it is that the margins of the opening are so uneven and jagged, and the compact tables bevelled from within outwards.

There is generally no elevation of the diseased spot above the level of the bone, or at most it is very slightly raised, yet I have observed that the growth which fills the cavity in the bone does sometimes rise above the surface and form a tumor, which, in a flat bone, projects on both its sides. And especially when the morbid growth consists of a gelatinous fluid, it expands the tables of the bone, in the form of a bladder, and in that state is probably the disease first seen by Van Wy, and named by him Hydrosteon. It must not be confounded with cysts and cystoid disease of bone.

This form of cancer does not differ in its elementary composition from that of fibrous and medullary cancer; every variety indicated above, in the aggregation of the elementary parts—in consistence—is sometimes met with in the same individual.

There can be no question as to the cancerous nature of the disease; it is quite common to find it combined with a very extensive production of cancer in the internal organs.

The nidus in which cancer growths originate on the Haversian canals, the tissues lining the cells and medullary cavities of the bones, the medullary system generally; and it is from these points that the compression, the erosion of the bony substance by pressure, and the formation of skeletons in the morbid mass proceed. Cancer almost always originates in the diplœ, in cancellous bones and parts of bones, or in the medullary cavities."

## Figures and Tables

**Figure f1:**
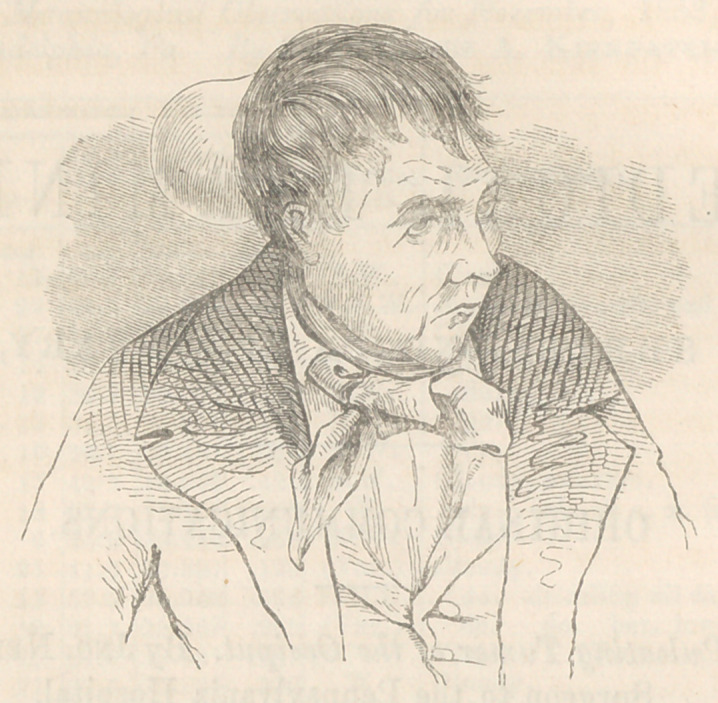


**Fig. 1. f2:**
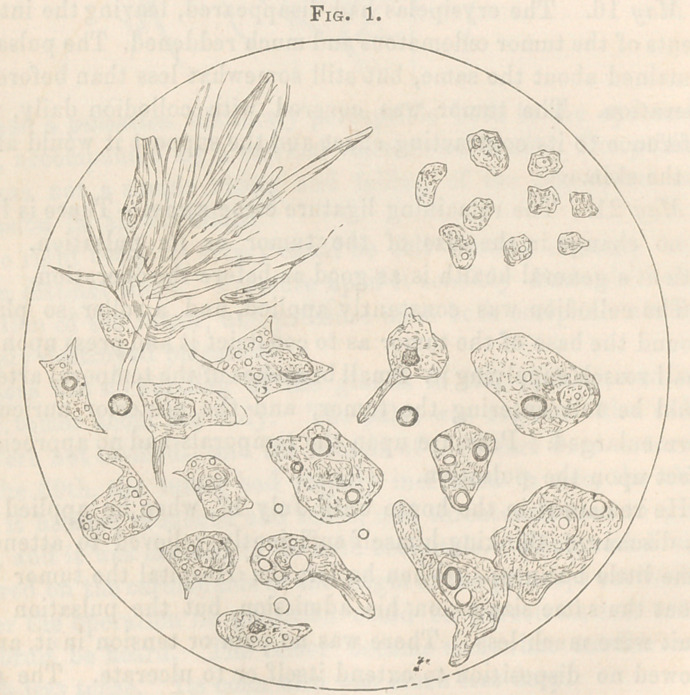


**Fig. 2. f3:**